# Design Principles for Manipulating Electrochemical Interfaces in Solid-State Supercapacitors for Wearable Applications

**DOI:** 10.1021/acsomega.1c00172

**Published:** 2021-03-18

**Authors:** Mihir Kumar Jha, Chandramouli Subramaniam

**Affiliations:** Department of Chemistry, Indian Institute of Technology Bombay, Powai, Mumbai 400076, India

## Abstract

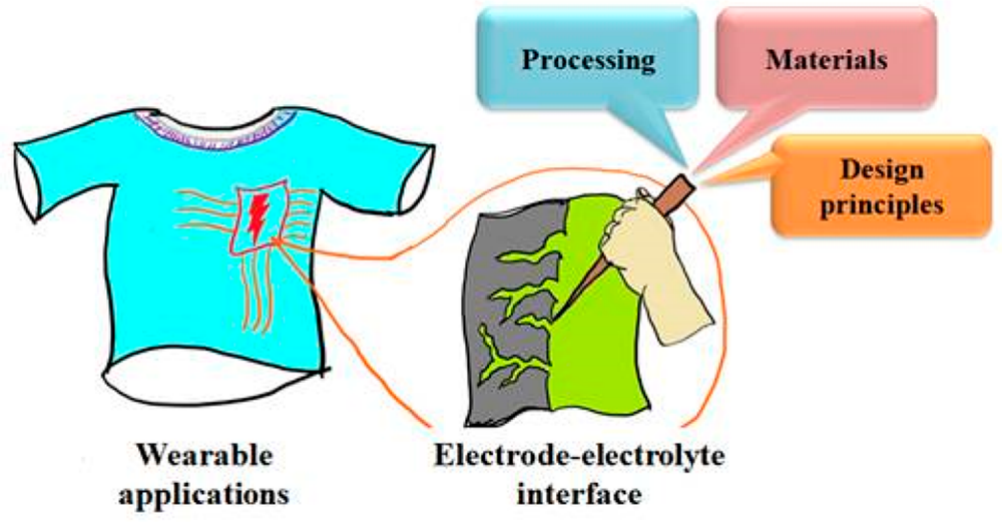

Storage and delivery of electrical energy form the heart of the rapidly expanding domain of wearable electronics, with applications ranging from point-of-care medical diagnostics to Internet-of-Things (IoT). Solid-state, electrochemical, double-layer-based supercapacitive energy storage devices, with high power density, ability to interface with intermittent energy harvesters, long lifetime, and cyclability, offer attractive possibilities for self-sustaining power sources in such portable applications. This mini-review highlights the need for a multipronged approach involving (a) development of materials for electrodes and electrolyte and (b) utilizing the right kind of design principles, processing techniques, and fabrication approaches to (c) achieve seamless all-solid electrode–electrolyte interfaces providing (d) facile integration onto wearable platforms. Importantly, a comprehensive figure-of-merit (FOM) accounting for both the electrochemical performance and the mechanical robustness of flexible supercapacitors is proposed. This is expected to facilitate uniform comparison of performance across devices differing in their design approaches and materials. Finally, new *operando* and *in situ* techniques for probing and understanding such all-solid interfaces are presented. The iterative cycle of scientific understanding, furthering technological advancements, seeks to provide future directions for achieving mechanically robust supercapacitors with enhanced energy density and power density for wearable and portable applications.

## 1 Introduction

Flexible supercapacitors (SCs) are increasingly becoming the preferred energy-storage platform for powering devices intended for portable applications such as Internet-of-Things (IoT) and point-of-care diagnostics. A major advantage with supercapacitors is their ability to interface with intermittent sources of energy that are accessible through energy harvesters.^1^ In this direction, flexible supercapacitors have been envisaged as the bridge between energy harvesters and batteries. Previously, research on flexible supercapacitors primarily dealt with the development of novel electrode and solid-electrolyte materials. Carbon nanotubes, graphene, metal oxides, conducting polymers, biomass-derived carbons, and other two-dimensional materials have been explored as electrode materials in flexible supercapacitors.^2^ Recent advances focus on configurational arrangement of the flexible components evidently because different design principles significantly affect the formation of the double layer.^3^ An ideal fabrication design for wearable electronics involves structurally flexible and mechanically robust components, without compromising their energy and power density. As opposed to a chemical battery that involves Faradaic reactions within the bulk phase of electrodes, the mechanism of capacitive storage is predominantly driven by the electrical double layer formed at the electrode–electrolyte interface. Therefore, it becomes mandatory to achieve a thorough understanding of such an electrode–electrolyte interfacial structure. The study of such electrified interfaces has witnessed significant evolution over the past 120 years, from the elementary model proposed by Helmholtz that was subsequently refined by the seminal contributions from Guoy-Chapman, Stern, and Bockris-Devanathan-Mullen. However, a comprehensive and unified model of the electrode–electrolyte interface still remains elusive, reaffirming the statement of Sir Wolfgang Pauli: “God made the bulk; surfaces were invented by the devil.”

Efforts to understand the electrified interface have witnessed resurgence in recent years, driven by the use of nanomaterials, with engineered porosity and high specific surface area as electrodes, for developing efficient energy storage devices with high energy density, high power density, and longer lifetime. Subsequently, there is no linear correlation between specific capacitance and pore diameter in the case of nanostructured carbon electrodes. The experimentally observed enhanced capacitance with micropores was conclusively attributed to the partial desolvation of the ions constituting the electrical double layer. This was subsequently reinforced by systematic theoretical insights aimed at deciphering capacitance–pore size correlations for versatile pore regimes.^4^ However, another major challenge while dealing with micropores was the reversibility of the EDL formation, which affects the cyclability of the device. This provides opportunities for the development of nanostructured electrode surfaces to achieve a balance of high capacitance along with greater cyclability.

**Figure 1:**
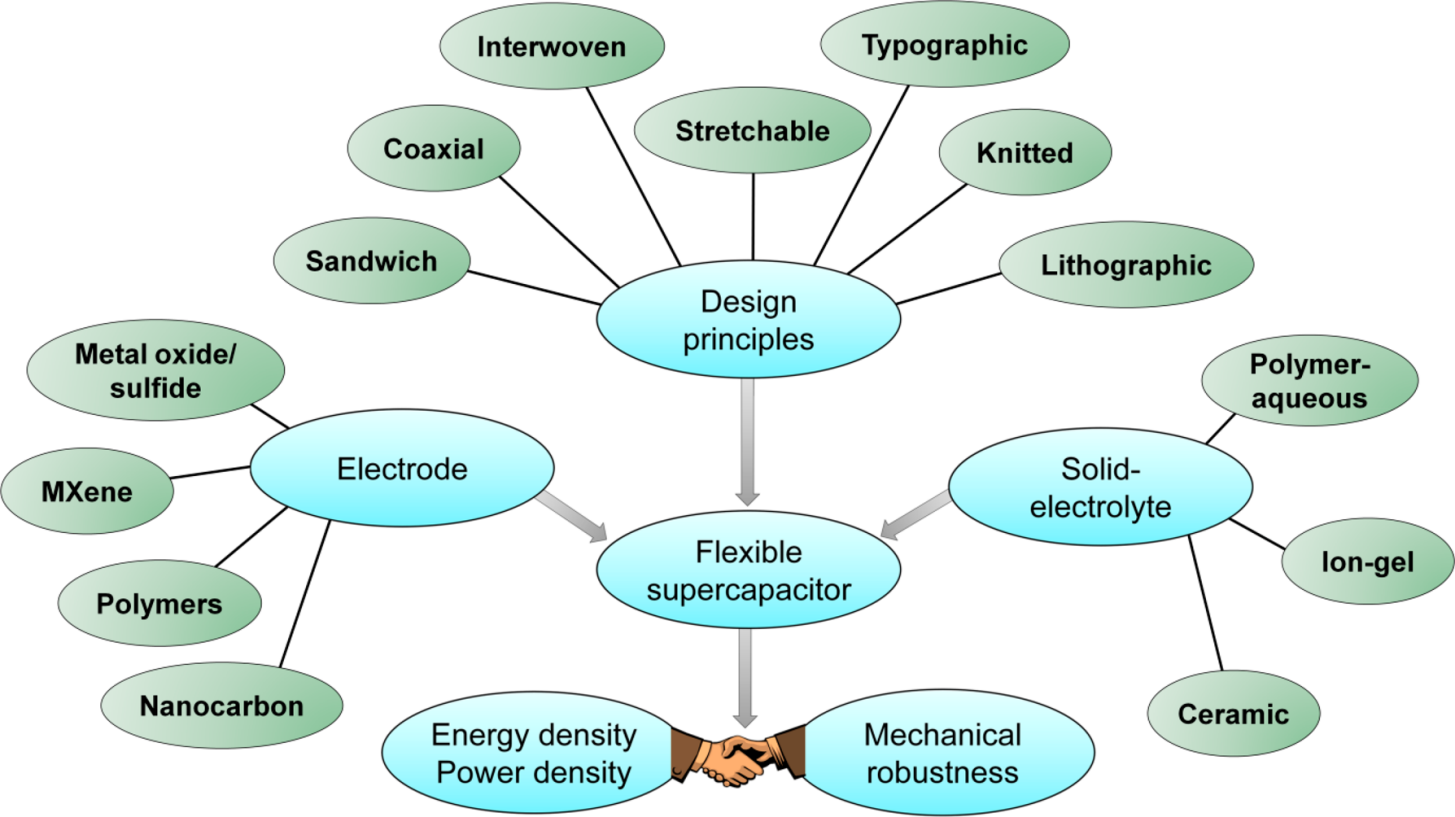
Path toward realizing supercapacitive energy storage devices for wearable applications.

Concomitant with deepening scientific interest, the field has witnessed technological developments that focused on the development of supercapacitive energy storage devices for powering portable electronics such as flexible sensors for monitoring vital physiological parameters, implantable medical diagnostics, smart devices, power T-shirts, and electronic textiles (Figure 1).^5^ Considering the rapidly expanding applications in the wearable domain, the power requirement range also varies across 6 orders of magnitude from microwatt to watt. In fact, the commercial wearable market is currently valued at USD 32 billion and projected to increase by 16% annually in the coming decade, triggering a demand for efficient and self-sustaining energy storage devices.

Accordingly, this mini-review intends to discuss the various aspects of design principles related to the development of solid-state supercapacitors. A qualitative and quantitative comparison of such design approaches, based on the technological viability and the demonstrated performance, will be presented. Furthermore, a universal figure of merit for comparing the performance of flexible supercapacitors is proposed. The discussion would also involve the use and development of newer materials for electrodes and electrolytes, in the context of wearable and portable electronics. Thus, this mini-review seeks to complement other exhaustive reviews written on materials for flexible supercapacitors.^6,7^ Importantly, this mini-review introduces the perspective of electrode–electrolyte interfaces in flexible supercapacitors and takes care of the additional manifestations of interfaces specific to “flexible” devices. In this way, performance metrics and mechanical requisites of flexible supercapacitors are described from the standpoint of interfaces.

## 2 Electrode and Solid Electrolyte Materials for Flexible Supercapacitors

**Figure 2:**
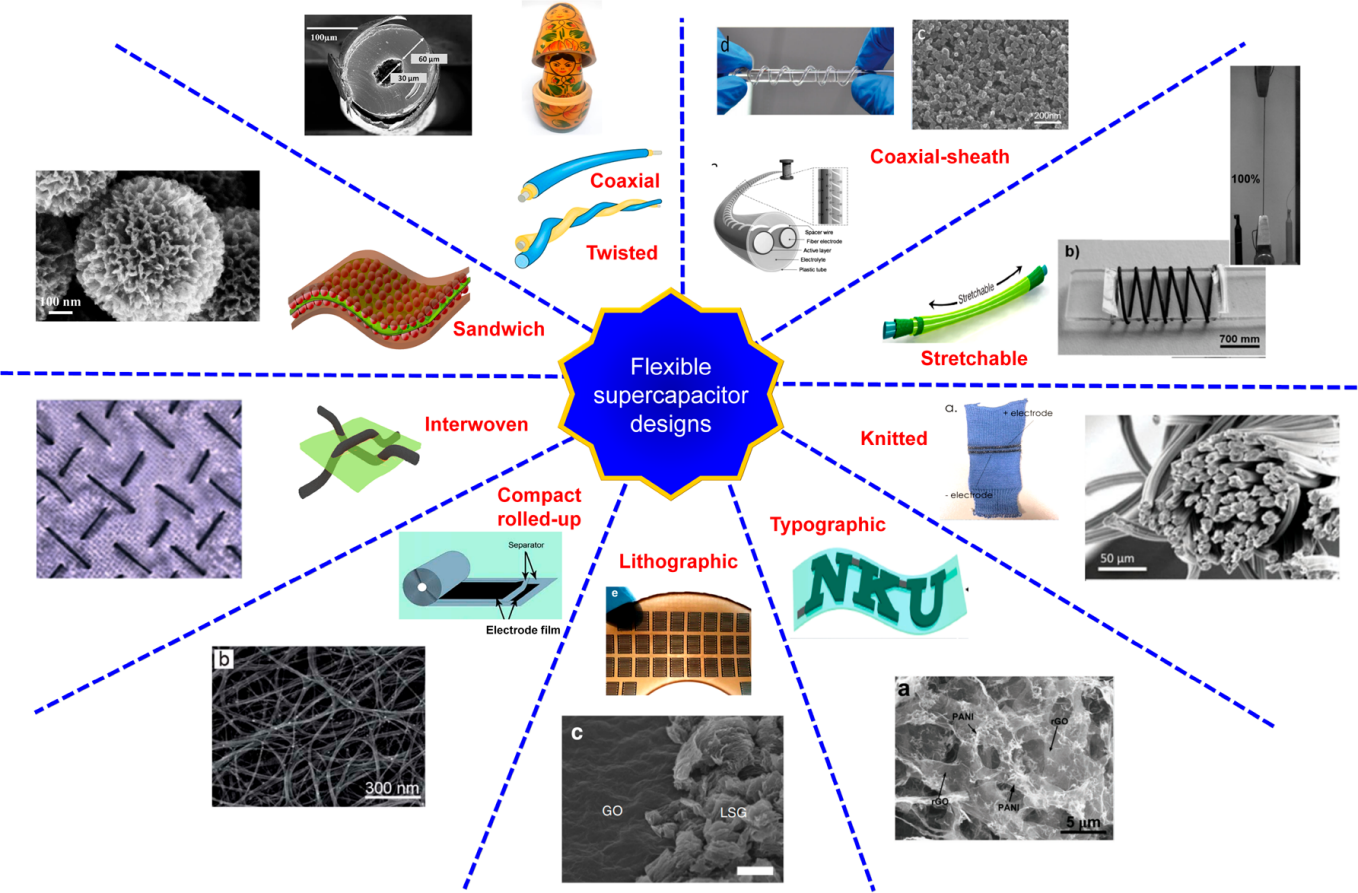
Illustrative schematics and electron microscope images.^9,11−18^ Images are adapted with permission from refs (9 and 11−18). Copyright 2019 American Chemical Society, Copyright 2020 American Chemical Society, Copyright 2017 Wiley, Copyright 2011 RSC, Copyright 2013 Wiley, Copyright 2018 Wiley, Copyright 2013 Nature Publishing Group, Copyright 2013 RSC, Copyright 2020 American Chemical Society.

The energy density of wearable supercapacitors can directly be correlated to the electrical conductivity, pore structure, and accessible surface area of the active electrode material in addition to its mechanical robustness. The first kind of electrode material, that holds promise for enhancing the energy density, is comprised of transition metal oxides and sulfides along with their carbon composites. Such compounds (MnO_2_, RuO_2_, IrO_2_, graphene-MnO_2_, CNT-MnO_2_, FeS_2_, Cu_2_S, Co_2_S_3_, and Ni_3_S_2_) exhibit rich electrochemical redox activity owing to the multiple oxidation states and the presence of unpaired valence shell electrons (Figure 2). In this direction, the combination of spinel/mixed oxide Zn_2_SnO_4_ (ZTO)-MnO_2_ on carbon microfibers was demonstrated to enhance the capacitance of the device.^6^ A similar improvement in the performance along with thermal stability is also observed while employing transition metal sulfides as electrodes. Pristine-conducting polymers, their doped variants, and carbon composites have been extensively studied as electrodes, primarily due to their high electrical conductivity, ease of solution processability, and mechanical tenacity that ensure seamless immobilization on flexible substrates including textiles. Composite fibers comprised of graphene and an organic semiconducting polymer (PPy, PEDOT, and PANI) exhibit high volumetric capacitance.^6^

A significant majority of the interest has focused on the development of nanostructured carbon materials as electrodes (Figure 2). One universal problem while dealing with nanocarbon materials is their strong cohesive van der Waals force, leading to their aggregation and thereby restricting its solution-state processability. Laser-scribed interdigitated graphene electrodes on flexible supports demonstrate high capacitance. Biomass-derived carbons are inexpensive and structurally diverse and offer versatile pore size distributions, suitable for energy storage applications. Moreover, the prevalence of abundant elemental doping provides a route toward achieving higher energy density (Figure 2).^6^ High volumetric capacitance is obtained with self-standing MXene films.^6^ The strength of solubility processability of MXenes provides versatility in design strategies for electrode fabrication, such as spray painting and patterned interdigitation.

Equally important to the performance of flexible supercapacitors is the choice of a solid electrolyte. Conventionally, aqueous-based electrolytes PVA-KOH, PVA-H_2_SO_4_, and PVA-H_3_PO_4_ are preferred because they are inexpensive and easier to synthesize. Despite the high ionic conductivity obtained (10^–3^–10^–4^ S/cm), a major limitation has been the inability of such electrolytes to operate beyond 1 V, due to possible Faradaic reactions involving water electrolysis. Accordingly, recent advances in solid electrolytes are therefore primarily focused on ion gels that can operate up to 4 V, thus opening up a possibility of integration with wireless transmitters and communication devices. For instance, Ahn et al. reported a flexible supercapacitor based on an ion-gel electrolyte operable at 4 V, based on 1-ethyl-3-methyl imidazolium bis(trifluoromethylsulfonyl)imide (EMITFSI) ionic liquid mixed with a cross-linked polymer.^6^ Furthermore, ceramic-based solid electrolytes such as Li_2_S P_2_S_5_ glass ceramic electrolyte have been reported to design a flexible supercapacitor based on multiwalled carbon nanotube electrodes.^6^

## 3 Fabrication Design and Interfaces in Flexible Supercapacitors

Phenomenal advancements in the development of electrode and electrolyte materials have shifted the focus toward the importance of design approaches to integrate the electrode and electrolyte and achieve a functional interface. Importantly, the choice of the electrode and the adopted fabrication design primarily influences the creation of the electrochemical interface. Accordingly, identical electrode materials have been demonstrated to form differing electrode–electrolyte interfaces in varying design principles.^3^ For instance, CNT film electrodes have been demonstrated to operate with extremely low relaxation time constants in lithographically fabricated compact devices.^8^ However, identical CNTs have resulted in a wearable and washable device in interwoven wire configuration.^9^ Consequently, the fabrication design and the electrochemical interface are interdependent. Therefore, the search for an ideal interface is incomplete without the right choice of the electrode material and design principle.

This mini-review seeks to highlight the importance of various geometric factors and approaches in designing devices that result in the creation of novel electrode–electrolyte interfaces. For instance, a sandwich design results in planar interfaces. Interfaces in lithographically micropatterned electrodes enable greater charge polarization from the edges of interdigitated fingers.^10^ Moreover, double-layer formation across coaxially assembled flexible supercapacitors involves three-dimensional cylindrical interfaces. A comprehensive theoretical understanding of such interfaces with microporous structures has been reported.^4^ Interestingly, textile-based designs including knitting and interweaving enable longitudinal and transverse charge polarization, leading to a unique interfacial structure.^9^ Therefore, the fabrication design and the structure of the interface are completely interdependable.

The first design involves sandwiching a solid-electrolyte sheet with two planar electrodes or rolling up the components in a cylindrical form, similar to conventional parallel-plate capacitor geometry (Figure 2). A trade-off between energy and power density in these devices is partially overcome by the fabrication of coplanar interdigitated electrodes (Figure 2). While design approaches based on lithography and spray coating are well established for coplanar devices, typography, printing, and additive manufacturing constitute newer developments holding greater promise for scalable implementation (Figure 2).^3^ Lithography offers higher spatial resolution of patterning, leading to a higher packing density of devices within a given area/volume. In contrast, spray coating and roll-to-roll printing present greater versatility in terms of both the type of materials and the substrates that it can handle (Figure 2). In addition, direct spray coating, inkjet printing, and additive manufacturing offer scalability and the luxury of handling extremely flexible and unconventional substrates such as fabrics.

**Figure 3:**
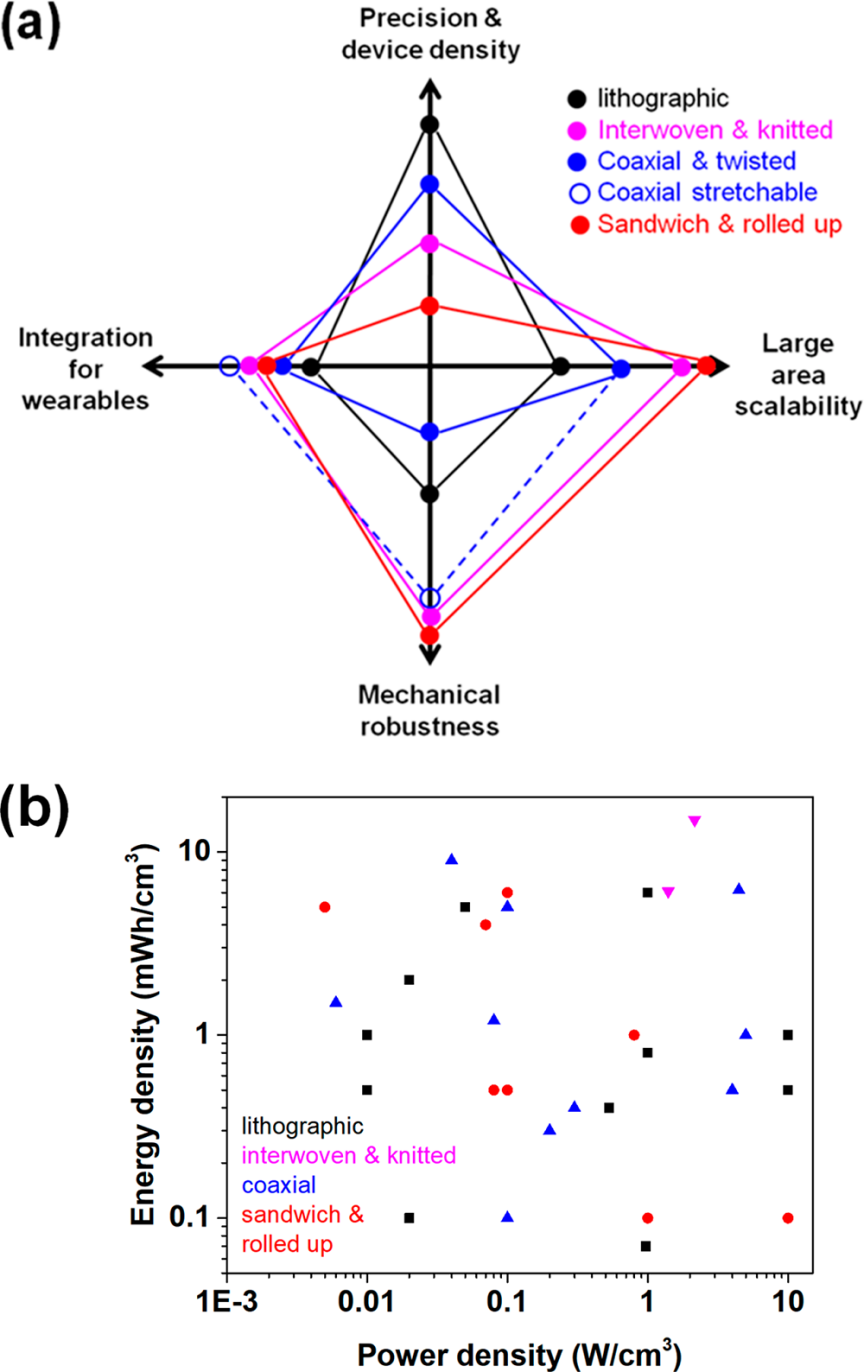
(a) Spider plot providing a qualitative comparison of the merits and challenges of various approaches in designing supercapacitors for wearable applications. (b) Quantitative comparison of the performance of flexible supercapacitors made using different design approaches.^7^

A tangential approach in this direction is to employ materials such as silk yarns and cellulosic threads as substrates and convert them to functional materials by uniform conformal immobilization of suitable nanostructured electrode materials. Accordingly, devices employing functional yarns and threads have been fabricated by interweaving, knitting, coaxial sheathing, and additive manufacturing routes (Figure 2 and Figure 3).^3^

An effective electrified interface has been demonstrated through a coaxial geometry that mimics the Russian *matryoshka* (Figure 2). The two electrodes can either be twisted in a predefined pattern or conformally cover each other with the dielectric material acting as the spacer doll. A modification of coaxial geometry involves twisting of two functional yarns within a matrix of gel electrolyte (Figure 2). Biscrolled yarns have been fabricated by inserting a twist onto a guest polymer sheet intertwined on a host wire.^3^ Such techniques result in effective utilization of the overall yarn porosity and accessible surface area, enabling applications such as ultrafast charging–discharging and AC-line filtering. Stretchable coaxial cable-type supercapacitors have been obtained by employing carbon–polymer composites, twisted yarns, and elastic substrates. A step ahead in frugal fabrication strategies has been achieved by creating microsupercapacitive junctions (sewcaps) with CNT wires.^9,19^ While sewcaps can store a sufficient quantum of energy, another dimension to the field is provided by the development of typographic and serpentine supercapacitors, where the electrode is designed to accommodate substantial mechanical duress during stretching or bending. Interesting designs among these involve the Japanese paper-art forms origami and kirigami to fabricate electrodes for flexible supercapacitors.^3^ A comprehensive summary of design attributes in flexible supercapacitors is outlined by Kaner and co-workers.^3^ Hence, reiterating Pauli’s statement, an effective interface can be achieved by manipulating and choosing a perfect design for fabrication of the device.

This aspect is more important for flexible and wearable applications, where mechanical duress experienced by the device is significantly higher than conventional applications. Therefore, formulation of design principles assumes greater significance in decoding the best configuration possible for achieving an effective electrode–electrolyte interface. Therefore, we attempt to compare the various strategies based on two different parameters, namely:

(a) a qualitative comparison based on aspects such as scalability, device packing density, ease of integration into wearable platforms and mechanical robustness that determine its technological viability (Figure 3a), and
(b) quantitative comparison based on the energy density and power density, as described by the Ragone plot (Figure 3b).

In this context, we have identified four crucial parameters, in addition to energy and power density. These are precision and device density, scalability, wearability, and mechanical robustness. Subsequently, a *qualitative* estimate of these parameters for different device designs is presented in the form of a spider plot. Such a *qualitative* estimate is evaluated from the performance of devices already reported in the literature. For instance, a device fabricated using lithography will always have the highest precision in device density. Moreover, coaxially assembled stretchable supercapacitors closely mimic clothing fabrics and are the most suited design for integration to wearable platforms. Furthermore, the integration of nanostructured materials on flexible supercapacitors comprised of lithographic micropatterning is intricate. This limits their relevance in industrial applications. Accordingly, industrial technologies encompassing roll-to-roll and inkjet printing, interweaving, additive manufacturing, and knitting are essential for seamless scalability. In addition to scalability, a comprehensive comparison is established with precision, device density, wearability, and mechanical robustness as the other parameters. Moreover, a *quantitative* performance descriptor has been discussed using the Ragone plot (Figure 3b), which includes the energy density and power density of devices fabricated with different design principles. The insights have been presented in Figure 3 and have clearly been captured from relevant literature reports.^2^ The scalability of a fabrication approach indicates its scope for extending the dimensions in order to satisfy the power consumption spectrum (Table 1). Consequently, interwoven or knitted designs are far more amenable to large-area scalability than lithographically patterned devices.

**Table 1:** Scalability of Devices Fabricated Using Different Fabrication Designs

design	power consumption spectrum^20^	power density (approximate values extracted from Figure 3)	dimension (cm^3^)
lithographic	μW–W	0.01–10 W/cm^3^	10^–4^–0.1 cm^3^
interwoven and knitted	μW–W	10^–6^–1 W/cm^3^	1 cm^3^
coaxial	μW–W	0.1–1 W/cm^3^	10^–5^–1 cm^3^
sandwich and rolled up	μW–W	10^–6^–1 W/cm^3^	1 cm^3^

Such a comparison seeks to present and correlate the design principle to the effectiveness of the electrode–electrolyte interface in terms of the performance metrics of energy storage and mechanical robustness. Therefore, this analysis would provide future directions for the selection and development of appropriate design approaches that not only are applicable to supercapacitors but also can be extended to other energy storage systems within the purview of wearable electronics.

The emphasis on the interfacial engineering by adopting newer and novel designs has gained increasing prominence. This has necessitated a universal parameter that can form the basis for comparing and evaluating the performance of devices that differ widely in both the material aspect and design principles. Therefore, a comprehensive performance-comparison parameter that captures both the electrochemical performance and its mechanical flexibility has been challenging, particularly because of the absence of universal guidelines. Improvements in the materials for electrodes and electrolytes are reflected as enhancements in the electrochemical parameters such as energy density and charge–discharge cyclability. Since the energy density forms the Achilles’ heel for supercapacitors, any advancement in materials would cause a strong impact on its energy density. We attempt to capture these aspects, encompassing the energy density, lifetime, reliability, and cyclability of supercapacitors by defining an electrochemical quotient (EQ) as
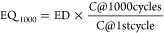
where ED denotes the energy density of the device, and C@1000 cycles and C@1st cycle represent the capacitance after the 1000th and first charge–discharge cycles, respectively.

Concurrently, the design has a direct bearing on the electrode–electrolyte interface especially under conditions of mechanical duress. Thus, the mechanical quotient is defined as
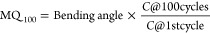
where C@100 cycles and C@1st cycle indicate the capacitance of the device after the 100th and the first bending cycles, respectively.

Quantifying the performance and electrochemical stability of supercapacitors forms the basis of EQ. Although EQ_1000_ captures this for up to 1000 cycles, it is potentially extendable beyond that to compare across a wider data set. Similarly, the mechanical stability under bending has been a widely used descriptor for flexible and wearable supercapacitors. Here again, a comparison to illustrate its efficacy has been made for 100 cycles of bending, with the scope of this being amenable for use beyond that. We envisage that EQ_*n*_ and MQ_*n*_, where *n* represents the cycle number, would form widely acceptable and comprehensive parameters that together capture the electrochemical advantage and mechanical robustness of the device in the context of flexible and wearable applications.

**Figure 4:**
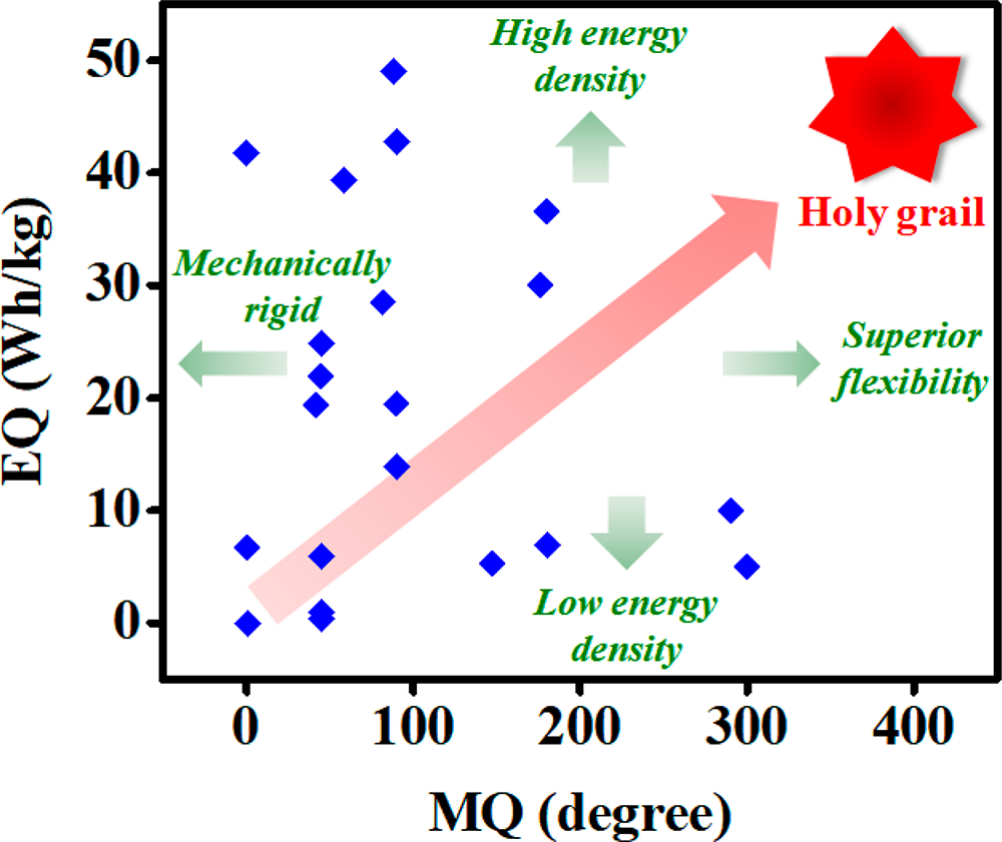
Figure of merit for flexible supercapacitors, demonstrated by comparing the electrochemical performance of devices with their mechanical quotients.

The validation of EQ_1000_ and MQ_100_ is carried out by adopting values from several literature reports in the form of an Ashby plot (Figure 4). Such a plot clearly distinguishes mutually exclusive domains that contain either high performing devices with low mechanical rigidity or devices with comparatively lower performance but with higher mechanical flexibility. Importantly, such a plot also lays down future demands and challenges in achieving devices that combine superior electrochemical performance with high mechanical flexibility, as pointed out by the diagonal arrow in Figure 4. This arrow signifies the figure-of-merit (FOM) with units of Wh/kg per degree and forms the holy grail of flexible and wearable devices. Notably, such a figure-of-merit is restricted to bendable devices and is inapplicable for stretchable supercapacitors, wherein an additional quotient for stretchability should be introduced. However, the proposed FOM serves as a basic formalism and descriptor for evaluating flexible energy storage devices. Similar principles can be extended to derive suitable FOMs for 10^3^, 10^4^, and 10^5^ bending cycles. We envisage that a similar FOM for stretchable supercapacitors should involve Poisson’s ratio of the electrode and electrolyte material, in order to evaluate its stretchability. Such conjectures are however a subject of future endeavors.

**Figure 5:**
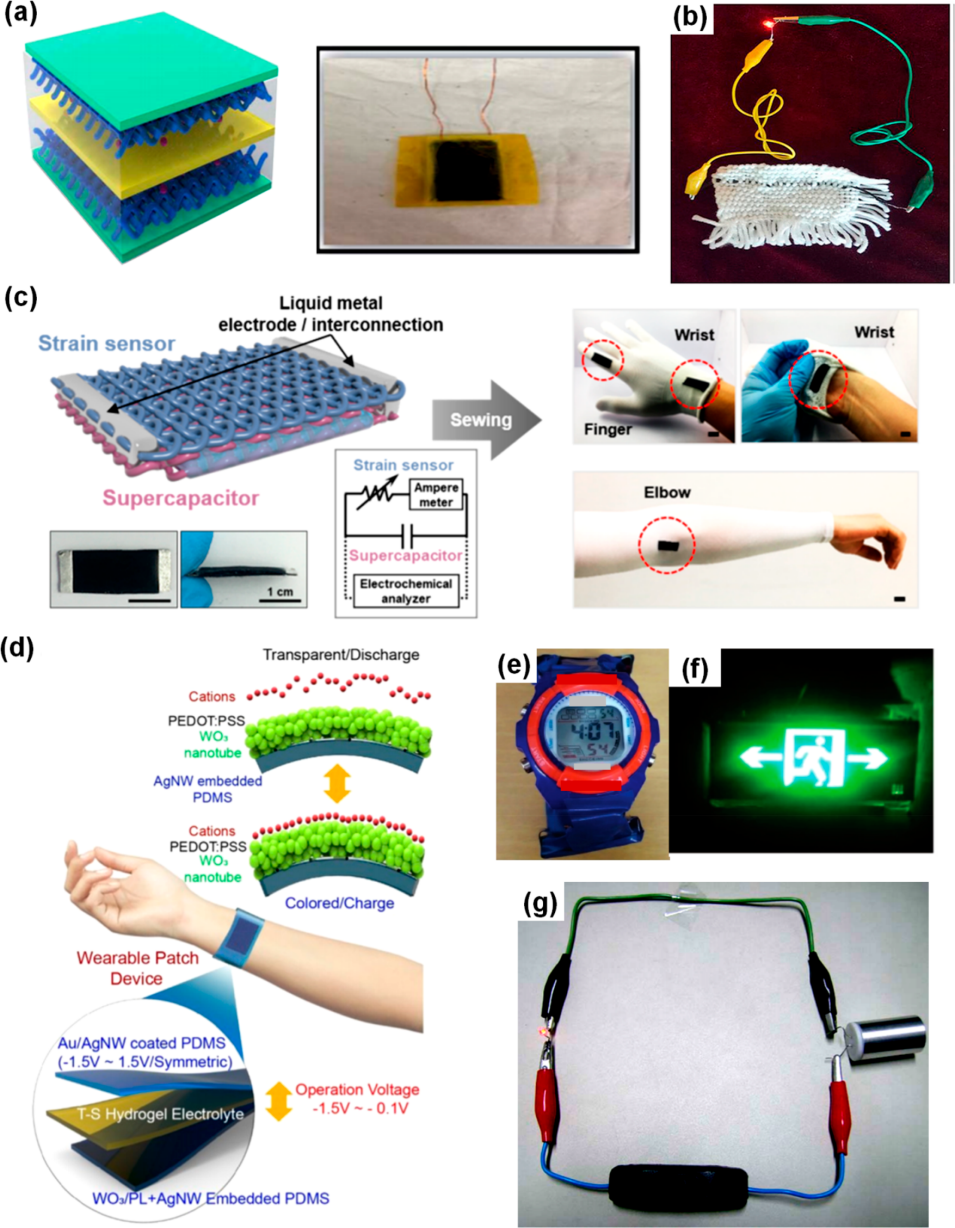
Applications of wearable supercapacitors. (a) Sandwich configuration in a symmetric piezo supercapacitor (left). Functionalized carbon-cloth-based electrodes (green) are arranged in sandwich geometry with a polarized PVDF film (yellow). The piezoelectricity thus generated is harvested to charge the supercapacitor. Picture showing the piezo supercapacitor (right). Adapted with permission from ref (21). Copyright 2015 RSC. (b) Powering an LED with a knitted fabric comprised of an integrated graphite-fiber-based supercapacitor.^22^ Adapted with permission from ref (22). Copyright 2017 Elsevier. (c) Schematic illustration and optical images of the fabricated strain sensor and supercapacitor with a circuit diagram of the integrated system (left). Integrated all-in-one system sewn onto a T-shirt and a nylon glove. The energy derived from the supercapacitor drives detection of biosignals by the strain sensor.^23^ Adapted with permission from ref (23). Copyright 2019 American Chemical Society. (d) Schematic illustration of an all-transparent stretchable electrochromic supercapacitor integrated in a wearable patch device.^24^ Adapted with permission from ref (24). Copyright 2019 American Chemical Society. (e) An electronic watch and (f) lighting of safety indication arrow comprised of 10 LEDs using a carbon-fiber-fabric-based large-sized wearable supercapacitor. Adapted with permission from ref (12). Copyright 2017 Wiley. (g) Image showing a compact-design supercapacitor lighting a red LED. Reproduced with permission from ref (13). Copyright 2011 RSC.

Applications of supercapacitors in wearable electronics are manifold. Interestingly, the charging potential of a supercapacitive device can be derived from piezoelectric polyvinylidene diflouride (PVDF) thin films (Figure 5a). Such concepts point toward the development of self-powered energy storage devices, wherein the harvested piezo voltage drives the charging of the storage platform. Moreover, coaxial wire-shaped supercapacitors are woven onto fabrics to power clothing. This has been demonstrated by powering an LED in Figure 5b. An important application of flexible supercapacitors lies in biosensing. Flexible supercapacitors have extensively been employed to power electrochemical sensors for human motion detection (Figure 5c). Generally, flexible devices are affixed onto muscular joints to demonstrate their comprehensive mechanical ability to bend, stretch, and flex (Figure 5d). The practical applicability of wearable supercapacitors has been exhibited by powering electronic watches, LED lighting, and patterns (Figure 5e–5g).

## 4 Emergent Directions toward Investigation of Flexible Interfaces

Although electrode–electrolyte interfaces have extensively been investigated through spectroscopic as well as microscopic techniques, no reports have specifically dealt with interfaces in “flexible” supercapacitive devices. This is because the fundamentals behind interfacial ion diffusion in electrical double-layer-based systems are equivalent across any electrode–electrolyte interface. A major impediment lies in the intricacy in probing flexible devices directly under a microscope or in a spectrometer. Therefore, such studies on interfaces are always restricted to cross sections of planar supercapacitors. In addition, design principles of flexible supercapacitors have rarely been studied from the standpoint of electrode–electrolyte interfaces. Accordingly, only planar sandwich models are taken into consideration for such studies. While interfacial phenomena across interfaces are equivalent, supplementary manifestations of diverse designs are broadly suppressed by such investigations. For instance, conventional analysis cannot examine the infiltration of the solid electrolyte in coaxial or yarn-based designs. Fundamentally, there are two main investigative routes for exploring the electrode–electrolyte interfaces in flexible supercapacitors.

**Figure 6:**
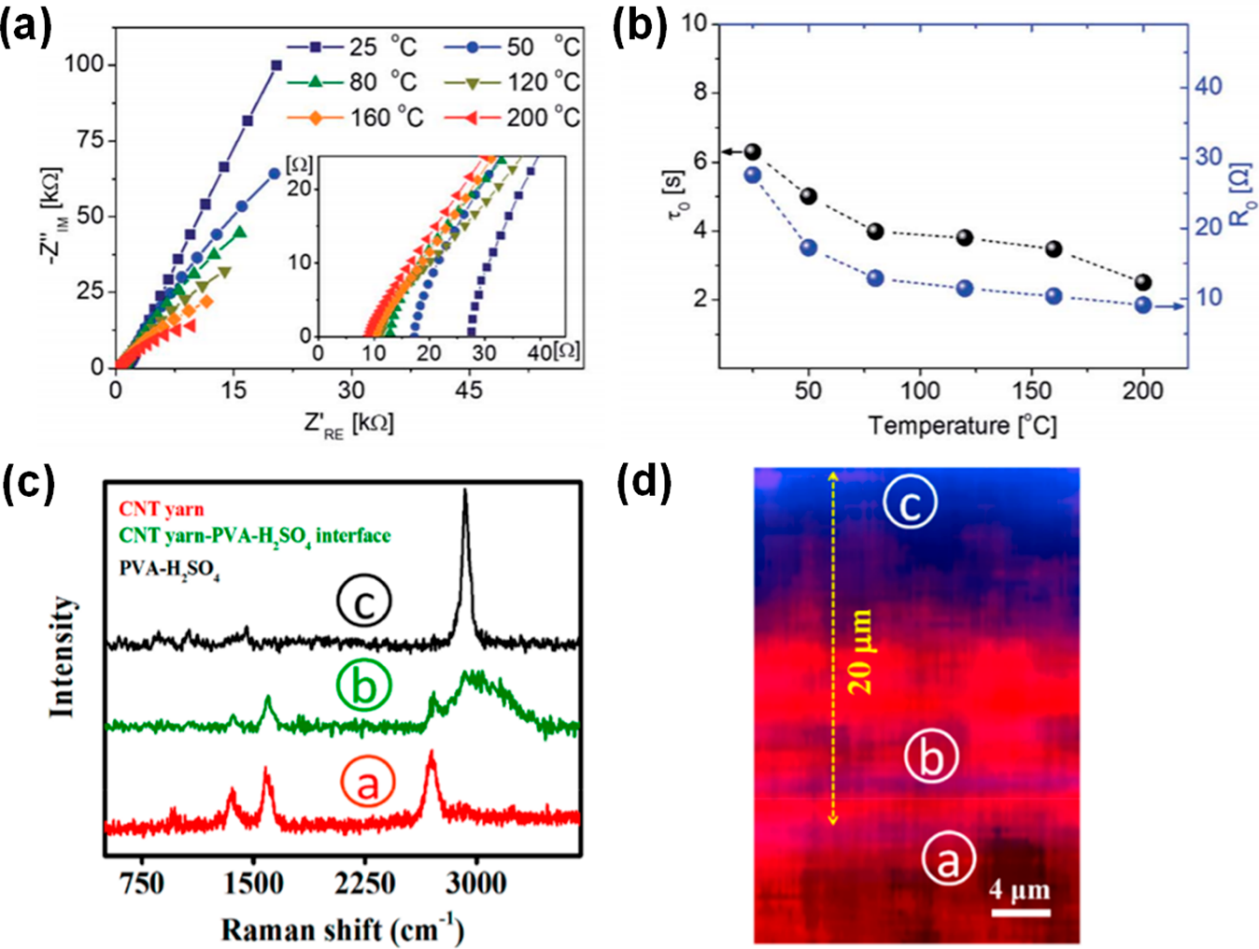
(a) Electrochemical impedance spectroscopy of a thermostable supercapacitor as a function of temperature. Nyquist plots of the multifunctional supercapacitor from 25 to 200 °C. Inset shows the data for the high-frequency region. (b) Variation of resistance and relaxation time with varying temperature. Reproduced with permission from ref (25). Copyright 2020 RSC. (c) Comparative Raman spectra of locations “a”, “b”, and “c” (as shown in (d)) representing CNT-yarn, diffused layer, and PVA-H_2_SO_4_ electrolyte, respectively, for an annealed coaxial supercapacitor. (d) Raman image of the electrode–electrolyte interface of an annealed coaxial supercapacitor. Reproduced with permission from ref (18). Copyright 2020 American Chemical Society.

The first route employs conventional electrochemical techniques including electrochemical impedance spectroscopy (EIS) to examine interfaces (Figure 6).^19^ A low EIS value is indicative of spontaneous diffusion of ions facilitating swift electrical double-layer formation at the interface. This is further complemented by the dielectric relaxation time constant that provides a measure of the time of discharge of the supercapacitor. Interestingly, EIS allows complete deconvolution of frequency-dependent diffusive and kinetically controlled regimes of charge storage, thereby providing crucial insights into the activation energy of ions and the performance of the supercapacitor (Figure 6).^25^ The decrease in relaxation time constant at increased temperature is indicative of rapid ion mobility at higher temperature.

Second, the infiltration of electrolyte ions facilitating enhanced frequency response of a flexible coaxially assembled supercapacitor is investigated using Raman spectro-microscopy (Figure 6).^18^ Conventional electron microscopy techniques cannot be utilized to extract images of a coaxial interface due to their confined depth of focus. Accordingly, a Raman microscope with a high *Z*-resolution of 250 nm has successfully been employed to image the coaxial interface. Furthermore, a chemical perspective of the interface is recognized by subsequent Raman line-profiling studies. A critical drawback of this method is the diffraction-limited resolution of the Raman images that fails to capture intricate ionic and electrochemical parameters at the interface. Despite such limitations, this offers a first-hand insight into the electrode–electrolyte interface in coaxial supercapacitors. Further research into this direction can proceed deeper to a subdiffraction level investigation and understanding of the electrical double-layer-based interface.

An interesting benefit of these techniques lies in their versatility and scope of integration with other techniques. Accordingly, Raman has been employed in conjunction with electrochemical tools to examine interfaces for liquid-electrolyte-based supercapacitors and in catalysis. Similar measurements were also recorded with X-ray diffraction and synchrotron studies. Moreover, temperature-dependent studies can also be carried out to infer the role of interfacial ion dynamics. Therefore, there is a broad scope of employing in situ operand measurements to examine events at the electrode–electrolyte interface.

## 5 Conclusions

This mini-review seeks to encompass the recent and significant developments in the rapidly advancing field of wearable, solid-state supercapacitors. These are presented in the context of development in materials for electrodes and electrolytes. On the scientific aspect, the performance achieved through various processing routes and design principles toward delivering a seamless and mechanically robust electrode–electrolyte interface is quantitatively discussed through a Ragone plot. A novel universal figure of merit for comprehensive comparison of flexible supercapacitors has been established. Simultaneous discussion on the technological benefits of the various design approaches and the critical role of form factor in governing the device integrability into wearable platforms has been qualitatively surveyed. Thereby, this mini-review seeks to provide (a) newer directions for understanding the electrified interfaces through (b) rational selection of material, processing route, and design approach, for furthering the applicability of supercapacitors in portable and wearable electronics. Techniques complementary to electrochemistry such as Raman spectroscopy and atomic force microscopy-infrared spectroscopy (nano AFM-IR) are currently being pursued to track and understand the real-time changes at the electrode–electrolyte interface. In combination with theoretical modeling, it should be possible to decipher the process leading to double-layer capacitive charge storage at the interface of nanoscale electrodes and electrolytes. An important challenge in this direction is to extend this for solid–solid electrode–electrolyte interfaces that form the critical part of wearable energy storage devices. Further, the effect of various design principles, discussed in this mini-review, on such interfaces and the opportunity to utilize thermal and chemical engineering approaches to extract the best out of the interface are a promising way forward in this domain. We envisage that this fundamental understanding would enhance our capability for a rational choice of the electrode material and the appropriate device fabrication approach.

## Dedication

This mini-review is dedicated to Prof. T. Pradeep, IIT Madras, on being recognized by the government of India with the Padma Shri award for 2020.
